# State of the Art for Microhaplotypes

**DOI:** 10.3390/genes13081322

**Published:** 2022-07-24

**Authors:** Kenneth K. Kidd, Andrew J. Pakstis

**Affiliations:** Department of Genetics, Yale University School of Medicine, New Haven, CT 06520, USA; andrew.pakstis@gmail.com

**Keywords:** microhaplotype, SNP, forensic genetics, individualization, ancestry, kinship, mixture deconvolution, paternity index, random match probability

## Abstract

In recent years, the number of publications on microhaplotypes has averaged more than a dozen papers annually. Many have contributed to a significant increase in the number of highly polymorphic microhaplotype loci. This increase allows microhaplotypes to be very informative in four main areas of forensic uses of DNA: individualization, ancestry inference, kinship analysis, and mixture deconvolution. The random match Probability (RMP) can be as small as 10^−100^ for a large panel of microhaplotypes. It is possible to measure the heterozygosity of an MH as the effective number of alleles (A_e_). A_e_ > 7.5 exists for African populations and >4.5 exists for Native American populations for a smaller panel of two dozen selected microhaplotypes. Using STRUCTURE, at least 10 different ancestral clusters can be defined by microhaplotypes. The A_e_ for a locus is also identical to the Paternity Index (PI), the measure of how informative a locus will be in parentage testing. High A_e_ loci can also be useful in missing persons cases. Finally, high A_e_ microhaplotypes allow the near certainty of seeing multiple additional alleles in a mixture of two or more individuals in a DNA sample. In summary, a panel of higher A_e_ microhaplotypes can outperform the standard CODIS markers.

## 1. Introduction

**Figure 1:**
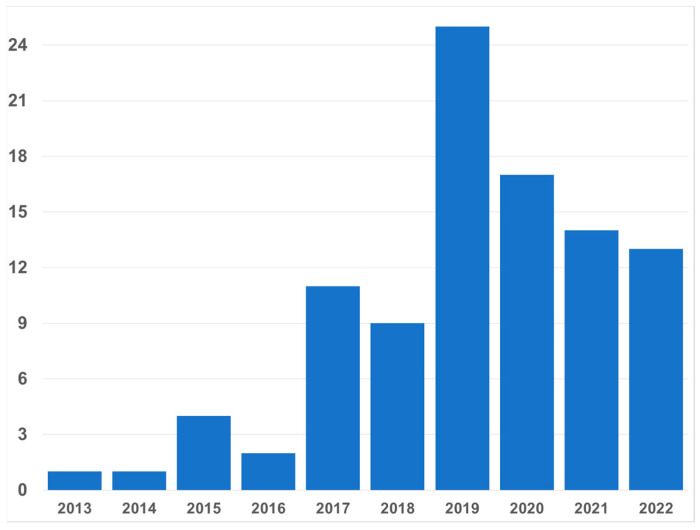
Yearly distribution of forensic papers about human microhaplotypes since the first in 2013 through 25 June 2022. Note that 2022 is outpacing 2021 so far. All fields were searched for “microhaplotype” or “micro-haplotype” in PubMed and in the journal *Forensic Science International Genetics Supplemental Series,* which is not included in PubMed.

It has been almost a decade since the first papers on microhaplotypes (MHs) were published; MHs were defined as small genomic regions with two or more SNPs defining three or more haplotypes [1,2]. In recent years, as many laboratories became interested in MH, the number of papers per year has averaged over a dozen (Figure 1). Most of those papers occur in the forensic literature. Clearly, there is considerable interest in MHs in forensics. The value of the MH is grounded in the facts that there can be many different alleles at a locus, and that the mutation rate of SNPs is very low compared to STRs, the multiallelic markers routinely in use for forensic work. Although most studies of MHs define the loci using binary SNPs, some researchers have used non-binary SNPs to provide even greater heterozygosity [3,4]. Others have broadened the concept to include indels as part of a microhap [5,6]. Earlier papers on multiple indels did not restrict the size of the locus or the number of alleles, but many papers from as early as 2013 might have included loci that fit the definition of a microhaplotype. More recent papers on multi-indels do not reach high levels of polymorphism.

In recent years, various research groups have reported hundreds of MH loci. Over 400 distinct microhaplotype loci are already included in the MicroHapDB [7], and this number will increase as loci from recent papers are added. A separate database effort, D-SNPsDB [8], categorizes all two SNP haplotypes in the 1000 Genomes (1KG) data. In addition to there being an increased number of published multi-SNP microhaplotypes, some recently published MH loci have high effective number of alleles (A_e_), a statistic used as a measure of how polymorphic the loci are [9]. The best published microhaplotypes range from an A_e_ around 3 to an A_e_ as high as 10 and above: see, for example, Wu et al. [10], Gandotra et al. [11], and Fan et al. [12]. This increase in highly informative microhaplotypes is ongoing and impacts all areas of forensic applications. 

**Figure 2:**
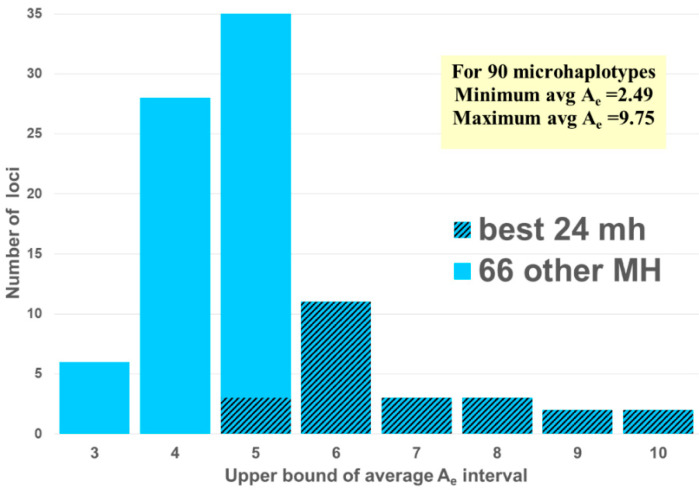
The distribution by the average A_e_ values of the 90 MHs [11,14] with the 24 MH [13] matched to the common STRs in [13].

A_e_ can be a proxy for how well MHs will perform. In fact, one small set of 24 microhaps with A_e_ values ranging between 5 and 10 provides more forensic information than the 24 commonly used STRs [13]. That study paired the top 24 MH from among the 90 MH studied earlier [11,14] (Figure 2) with the 24 most commonly studied forensic short tandem repeat (STR) loci. In all compared areas, the MHs were better than the STRs. Thus, the issues in studies now relate to the value of specific MH loci in specific areas of the world because of global variation in MH allele frequencies.

Here, we consider briefly the state of the art in each of four main areas of forensics: individualization, ancestry inference, kinship analyses, and mixture deconvolution.

## 2. Individualization

**Figure 3:**
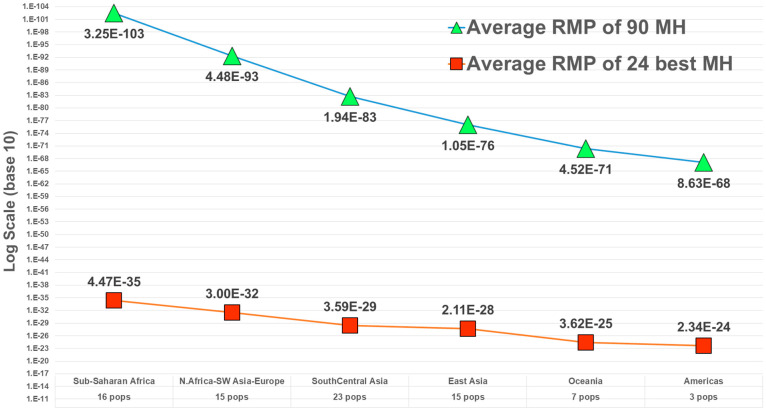
Average RMP in major world regions for a dataset of 90 MHs and 79 populations (from [14]) and for the best subset of 24 MHs [13].

**Figure 4:**
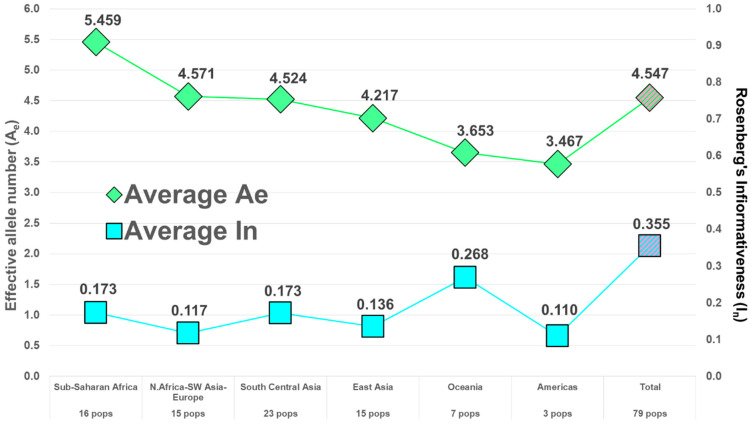
Average A_e_ and average I_n_ in 6 major world regions for 90 microhaplotypes.

**Figure 5:**
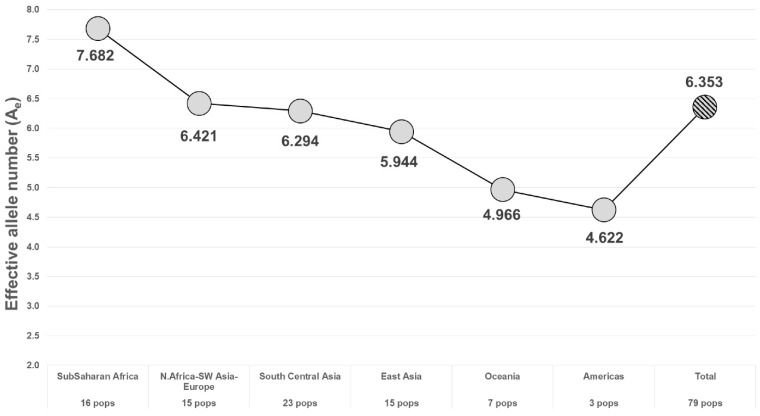
Average A_e_ of the 24 best MHs in 6 world geographical regions.

Following the example of the first uses of DNA in forensics, among the first and most common uses of microhaplotypes has been determining whether an evidence profile matches a suspect’s profile. The random match probability (RMP) (also known as cumulative match probability, CMP) of finding a match of an unrelated individual to a target person evaluates the probability statistically. The lower the RMP, the less likely the match is a chance event, and it is relatively more likely that the match means the evidence came from the suspect. By selecting many microhaps, it is possible to have a RMP of slightly less than 10^−100^ [14] in some populations (Figure 3). The commonly studied forensics markers include 24 short tandem repeat (STR) polymorphisms, and the probability of a random match for large populations is about 10^−30^ or slightly larger [13]. As MHs with higher A_e_ values are being found, it is more evident that very small RMPs are possible with even fewer loci than the full 90 MH panel [14]. As an exercise in comparing the 24 most commonly used STR polymorphisms with microhaplotypes, Kidd et al. [13] chose the 24 highest A_e_ loci in the dataset of 90 microhaplotypes [14] (Figure 2). That set of microhaplotypes yielded slightly better (smaller) RMPs than the CODIS STRs routinely used in forensics. Figure 3 shows the average RMP valuesof those 24 microhaplotypes in six major world geographical regions while Figure 4 and Figure 5 show the average A_e_ in those regions for the 90 MH and the 24 MH datasets. The inclusion of any additional loci with A_e_ of 5 or higher would enhance the superior performance of microhaplotypes over the enhanced CODIS markers.

The RMP for different regions of the world shows a commonly seen pattern of less heterozygosity as one looks at populations farther from Africa. This is reflected in most studies of SNPs and of microhaplotypes (e.g., [15,16]). Whatever the cause, there is some potential counter effort, since many studies are ascertaining larger numbers of MHs using East Asian populations (e.g., [10,12,17]). Thus, more MHs with higher A_e_ values in East Asian populations are becoming available. While these studies focused on other aspects of MHs, usually mixture deconvolution, the higher A_e_ values of the markers they published, based on the scanning of whole genome sequence (WGS) data such as the 1000 Genomes (1KG) data, indicate a high value for individualization. There is a potential issue of ascertainment bias, with loci being searched for in Chinese populations, which is illustrated by the study of Zou et al. [18], as discussed below.

## 3. Ancestry Inference

Ancestry inference refers to determining the population origin of a person. This is distinct from kinship analysis (see below). The statistic that measures the relative value of markers for ancestry inference is informativeness (I_n_) [19]. STRUCTURE [20] or ADMIXTURE [21] and principal components analysis (PCA) are two approaches that are commonly used with ancestry informative markers (AIMs) to illuminate population relationships. The logic is that as individuals from the same population cluster together to the exclusion of other population clusters, the more likely the dataset will assign an unknown value to its proper population when reference data are available for that population. In practice, whichever AIMs are used, closely related populations are difficult to distinguish. 

Since 2006, many panels of single nucleotide polymorphisms (SNPs) have been used to infer the ancestry of individuals by showing which populations can be grouped together [22,23,24,25]. A common finding is that six major groups of populations can be easily shown, the so-called continental populations: African, European, South Asian, East Asian, Native American, and Pacific Islander (Oceanian). New ancestry informative SNP panels continue to be published, although many of the more recent panels have been more focused on differentiating between populations in particular regions of the world. However, the state of the art has shifted to microhaplotypes because microhaplotypes are more informative than SNPs. Those SNPs, however, can be used in conjunction with MHs to increase informativeness for an area of specific focus in a study. For example, massively parallel sequencing (MPS) can include individual SNPs and MHs in the same run, as well as STRs [13].

The forensic STRs are poor at ancestry inference [13]. Many panels of SNPs have been proposed, but recently, panels of MHs have also been proposed. MHs have been used in combination with SNPs in some recent studies [26,27]. Two recent studies included large numbers of MHs and many populations [14,28]. More common are studies that have used the 1000 Genomes (1KG) data [29] to test the value of a panel. 

A problem is that 1KG is not a good global panel of populations, and the samples tend to be geographically clustered as African, European, South Asian, East Asian, and admixed American populations. Thus, most studies will reveal those clusters and not much, if any, resolution at the single population level, unless that population is an outlier among those being analyzed. They cannot show a clear Middle East cluster, a North Asian cluster, or a Native American cluster because reference populations in those areas are not present. Recent studies collecting population samples from a more uniform sampling of world areas make it possible to identify additional population clusters distinct from the previously identified world regions. One study [26] demonstrated a separate cluster of SW Asian/Middle East populations. Another [30] found a distinct North Asian cluster. More comprehensive studies of populations in the Americas, Oceania, and SubSaharan Africa should be able to reveal more complex regional clusters. 

Early studies using MHs showed the same patterns of population relationships that were shown by large SNP panels with broad geographic coverage [17]. However, the most recent panels of MHs allow more detailed groupings of populations. The panel of 90 MHs can identify at least seven different clusters when all 79 populations are analyzed by STRUCTURE. When biogeographic regional subsets are analyzed, much more detail is available [14]. For example, Sub-Saharan Africa subdivides into several clusters (see Figure 6 in [14]): Central Africa, two West African groupings along a west-to-east axis, and two clusters for East Africa. East Asia subdivides into three clusters (see Figure 7 in [14]) along a north-to-south axis. 

**Figure 6:**
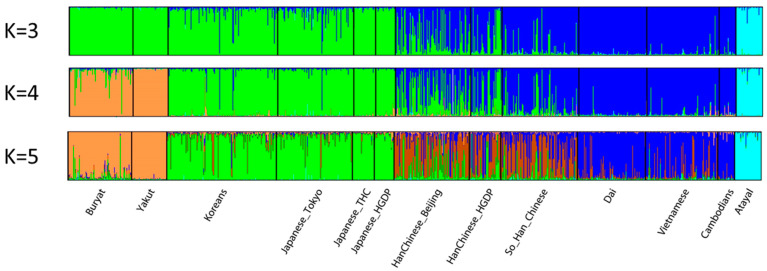
Individual bar plots of 13 East Asian population samples from STRUCTURE runs of the 90 MH dataset. This reanalysis with STRUCTURE was conducted by omitting other populations studied in [14]. The results of the highest likelihood runs at K = 3, 4, and 5 are shown.

Of the several recent studies of relationships among East Asian populations, the study by Zou et al. [18] is particularly interesting. They selected five “Chinese” and Japanese populations in the 1KG to screen for highly informative MH and identified 21 markers. They then genotyped those 21 markers on an independent set of nine East Asian samples. What they found was that the new populations were not as distinct as the original set of populations on which the selection of markers was based. This difference is indicative of an ascertainment bias. Moreover, the 21 markers provide no clear subdivision of their populations in the STRUCTURE analyses, although PCA showed a better dispersal of the East Asian populations than European populations. In contrast, STRUCTURE analyses using the 90 MH loci on just the 13 East Asian populations (omitting all other populations) (Figure 6) shows a highly “admixed” pattern for the Han Chinese samples, analogous to that seen for all of the Asian populations in the results of Zou et al. We include this example to show a high heterogeneity among the individual Han Chinese.

## 4. Kinship Analysis

In contrast to ancestry inference, which tries to reconstruct populations and their relationships, kinship analysis attempts to evaluate biological relationships within families. The most commonly used kinship analysis is the paternity index (PI). It is used to calculate the likelihood ratio of the probability of a man and child sharing an allele if the man is the true father, divided by the probability of such sharing if a random man is the father. In most situations with highly polymorphic loci, the PI value for one locus is 0.5, divided by the population frequency for the shared allele at that locus. The average PI value for a locus is 0.5, divided by the sum over all alleles at the locus of the allele frequencies squared. This average is a constant (usually 0.5) times the definition of A_e_. Thus, highly polymorphic loci with a high A_e_ are also better in paternity tests than loci with a low A_e_. A_e_ can be considered a population specific measure of the value of a locus for paternity testing. Across multiple loci, the locus specific PI values can simply be multiplied. A combined PI of >>100 can be considered strong DNA evidence favoring paternity. Paternity testing is currently carried out with standard STRs in most parentage testing laboratories, but classical markers (e.g., blood groups, etc.) can also be used. We noted above that selected panels of MHs can have better A_e_ values than the STRs, and the use of MHs decreases the potential problem of mutation that occurs when using the STRs. Thus, the non-DNA evidence being the same, MHs panels at least as good as the 24-MH panel should be better for paternity testing than the current STRs. 

Kinship studies also include family reconstruction and testing for a biological relationship as in immigration and missing persons cases. A more distant relationship between two individuals can be shown to be likely if the marker is more heterozygous, i.e., the A_e_ value is higher. Unidentified human remains can be identified if the DNA profile fits a relationship in a family cluster. Again, we note that MH panels now being identified are more heterozygous and have a higher A_e_ than the standard forensic STR markers. Although not using a panel of the most heterozygous markers, even evidence favoring a third degree relationship has been shown with microhaplotypes [31]. A different large panel of 417 MH with an average A_e_ of 3.57 was not sufficient for first cousin testing, but a smaller panel with a higher average A_e_ of 4.76 was able to determine second degree relationships [32]. Panels of MHs with average A_e_ much higher than 5.0 are now available, so a reliable estimation of more distant relationships should be possible. However, SNP chips with >100,000 SNPs will probably not be equaled by MHs in their ability to identify distant relationships.

Non-invasive prenatal paternity testing is possible using fetal DNA in maternal circulation [33]. The large number of alleles of the more polymorphic loci provides a high likelihood that the fetus’s genotype differs from the maternal genotype. The size range for most MHs makes it likely that the full alleles at the MH loci will be recoverable. 

One of the assumptions is that there are no “mutations” involved in these kinship studies. The possibility that loci with high A_e_ also have high levels of recombination and/or mutation is a caveat that needs to be considered. If the PIs from nearly all loci are consistent with parentage and the cumulative value is sufficiently high, an individual locus exception is usually ignored. A single locus excluding parentage can be attributed to lab error, mutation, or recombination and is not sufficient for an overall exclusion.

## 5. Mixture Deconvolution

MHs are likely to be very important in the deconvolution of mixtures of DNA from two or more individuals. With the high sensitivity of current DNA typing methods, mixtures are frequently detected and need to be correctly interpreted. The advantages of analyzing microhaps compared to STRs are (1) the absence of stutter because there are no repeats that would allow polymerase slippage; (2) fewer stochastic effects, e.g., the elimination of preferential amplification of shorter alleles because both alleles are the same size; and (3) potentially increased robustness due to smaller amplicons, which will more successfully amplify degraded DNA. MHs can also be as polymorphic as, or even more polymorphic than, the STRs, as illustrated in the sections above.

The probability of fully resolving the mixture at a locus will be a function of the allele frequencies in the population. The desirable result for the analysis of a two person mixture (although it is not known in advance that the sample is a mixture, much less a two person mixture) is to see four different alleles in the genotyping results for a locus. It is only possible to see four alleles in a two person mixture if at least four or more haplotypes (alleles) exist in the population. If one of the components of a mixture is a known individual, seeing all four possible alleles adds the genotype of the unknown contributor to the mixture by simply excluding the alleles from the known individual.

The probabilities of seeing three, four, or more alleles at a locus as proof that a mixture exists are functions of the array of allele frequencies of the persons in the mixture. Estimates of the actual probabilities are best dealt with by simulation. However, an approximation can be estimated if the simplifying assumption of the integer effective number of alleles is used as an approximation to the allele frequencies in the population. For simplicity in the calculations, we used the immediately lower integer for each A_e_ value to give a minimum estimate of observing all four alleles in a two person mixture (cf. Table 1), considering a marker with A_e_ in the interval. That integer corresponds to the equivalent number of equally frequent alleles, and the inverse corresponds to the frequencies of those hypothetical equally frequent alleles. As the A_e_ increases, the number of combinations of four different alleles increases, even as the allele frequencies become smaller and the probability of seeing at least one locus with four alleles increases. These numbers can be used to calculate the probabilities of the various possibilities. The probability of seeing four alleles at one locus of the 24 loci (cf. Figure 2) is greater than 0.999 [13]. This is a conservative estimate based on using the lower bound of each A_e_ interval; the estimate using the exact A_e_ values within each interval would be larger, with each value closer to the value for the next higher interval. The results from actual mixture studies illustrate the value of the high A_e_ markers in this set of 24 MHs [13,34,35,36]. These examples are based on the SNPs originally used to define the loci (cf. ALFRED; https://alfred.med.yale.edu) for a panel of 74 MH loci and incorporated in the ThermoFisher software [36]. 

## 6. Future Directions

We noticed that the naming convention proposed by [37] is being inconsistently followed. Some studies (e.g., [18]) use the set of SNPs to rename some microhaplotypes because an additional SNP was identified in the region. In some cases, the locus name had already been assigned to define a molecular region. We prefer the convention of naming a molecular region, since it is consistent with the naming of human genes. It also avoids the issue of multiple different names that depend on the variants used for the same molecular region. As sequencing becomes common, different populations will likely have somewhat different sets of useful SNPs, which could lead to a plethora of names to the common locus. We hope future researchers will use the molecular region as the basis for a locus name.

We have proposed a set of 24 microhaplotypes to be more broadly studied with the explicit thought that the good should not be a hostage to the perfect [13]. Additionally, we see no evidence that in the near future any large number of high A_e_ markers is likely to be tested on as large a population sample—4010 individuals in 79 distinct populations. Even if a different set of microhaplotypes eventually becomes an agreed-upon forensic panel (a likely ultimate outcome), a commercial kit of the current 24 loci would allow more populations and larger sample sizes to be studied. Additionally, the kinks of using microhaplotypes in forensics could be explored. 

In the meantime, it is important to compare different sets of MHs on the same set of populations and individuals to standardize their A_e_ and I_n_ values. Without more standardization, it will be difficult to choose an overall optimum. Moreover, with standardization it may be possible to identify panels of MHs that are optimal for different regions of the world.

While one can estimate the probabilities of seeing different numbers of alleles given a known mixture and allele frequencies, the ultimate objective is identifying the components of an unknown mixture. Two person mixtures can be relatively easy to interpret with multiple high A_e_ markers. The examples just cited allow the user to make reasonable guesses as to the number of contributors and their relative amounts in more complicated mixtures. However, guesses are subject to subtle biases. More importantly, rigorous statistics are essential. A program for probabilistic genotyping analogous to those available and in common use for analyses of the CODIS STR markers [38], needs to be developed. Some level of standardization will also be necessary for development of probabilistic genotyping software. 

As researchers identify more MHs with high A_e_ and/or high I_n_, the question of stability of the region to intra-microhaplotype recombination arises. While we do not think this is an issue, except possibly in kinship analyses, it is a question that needs to be considered. With a genome average of about 1% recombination per megabase, recombination within a 200 bp–300 bp region will be comparable to mutation rates for SNPs. However, in the original definition of microhaplotypes [2], a criterion was that recombination hot spots should be avoided. That criterion has largely been ignored. However, loci with very high A_e_ values (e.g., >10) can only have arisen with high mutation rates, on average, across the region and high historical recombination to shuffle those variants. Thus, by selecting loci with very high A_e_, one is preferentially selecting such loci. How big this ascertainment bias is as applied to individual loci is a question for exploration. Pending studies to explore possible recombination, the integrity of very high A_e_ microhaplotypes already identified seems to be sufficient for all forensic analyses.

## Figures and Tables

**Table 1:** Lower bound probabilities of seeing 4 alleles at one locus in a two-person mixture (from [13]).

A_e_ Interval	Probability of 4 Alleles Being Present at One Locus	Number of Loci (n) in Interval for 79 Populations	Probability 1−(1−prob) ^n^
4 < A_e_ < 5	0.094	3	0.256
5 < A_e_ < 6	0.192	11	0.904
6 < A_e_ < 7	0.278	3	0.623
7 < A_e_ < 8	0.350	3	0.725
8 < A_e_ < 9	0.410	2	0.652
9 < A_e_ < 10	0.461	2	0.709
Cumulative Probability		24	0.9992

n refers to the number of loci.

## Data Availability

Data underlying various illustrations in this paper (Figure 2, Figure 3, Figure 4, Figure 5 and Figure 6) is freely available and was deposited to the Zenodo archive; for links to those submissions see data availability sections in [14,30].
